# How Early Is Too Early? Use of Lineage-Specific Chimerism in Early Detection of Donor-Derived Malignancy After Allogeneic Stem Cell Transplant: A Case Report

**DOI:** 10.1155/crh/8230549

**Published:** 2025-11-11

**Authors:** Victor Zaman-Pope, Adrienne Fulford, Uday Deotare, Shona Philip, Christopher Burnie, Angela Hardeman, Anargyros Xenocostas, Anahita Mohseni Meybodi

**Affiliations:** ^1^Department of Medicine, Division of Hematology, Schulich School of Medicine and Dentistry, Western University, London, Ontario, Canada; ^2^Pathology and Laboratory Medicine, London Health Sciences Centre, London, Ontario, Canada; ^3^Department of Pathology and Laboratory Medicine, Schulich School of Medicine and Dentistry, Western University, London, Ontario, Canada

## Abstract

Allogeneic stem cell transplant is critical for treatment of certain hematologic malignancies. However, it has significant risks including relapsed malignancy, infection, and graft versus host disease. Rarely, de novo malignancy can arise from donor cells. Chimerism analysis is used to monitor engraftment and predict rejection or disease relapse. Our patient underwent an allogeneic transplant for myelodysplastic syndrome but had persistent pancytopenia despite donor lymphocyte infusion. This was due to donor-derived malignancy, which was predicted by loss of a satellite marker on chimerism analysis 6 months prior. This could have allowed earlier intervention and underscores the importance of detailed chimerism monitoring.

## 1. Introduction

Allogeneic hematopoietic stem cell transplant (allo-HSCT) is a critical tool in the management of acute leukemia, myelodysplastic syndrome (MDS), and other hematologic malignancies. It offers the potential for long-term survival in conditions lacking curative therapy by leveraging donor-derived allo-immunity and graft versus leukemia effect [1]. As part of the procedure, patients are given conditioning therapy to allow for engraftment of donated hematopoietic stem cells. Peripheral blood chimerism analysis is then used to monitor engraftment kinetics and predict graft rejection or early disease relapse [2]. However, even with adequate engraftment, allogeneic transplantation continues to impose significant risks including relapse of hematologic malignancy, infection, and severe graft versus host disease (GVHD) [3]. The most common of these, relapsed malignancy (which occurs in approximately 30% of patients), confers a very poor prognosis, with median survival measured in months [3–5]. While the majority of malignancies post allo-HSCT result from recurrence of the original hematologic malignancy or from de novo secondary malignancy related to treatment, rare cases of leukemia arising from donor cells have also been reported [5–7]. The prevalence is difficult to determine as many are case reports; a European retrospective cohort estimated that donor-derived leukemia occurred at a rate of 0.8 per 1000 transplants [5, 6].

The first case report of posttransplant donor-derived malignancy in 1971 detected leukemic transformation in the male donor cells through karyotype analysis showing XY chromosomes in the female recipient [8]. Current methods for determining cell origin have evolved and no longer rely on sex discordance. Instead, highly polymorphic microsatellites with short tandem repeats and variable number tandem repeats are used to determine cellular origin [2, 4]. These techniques allow for both highly sensitive chimerism analysis and lineage-specific chimerism.

Here, we report an interesting case in which a loss of a satellite marker used in routine chimerism analysis detected chromosomal abnormalities in the donor cells arising months before overt donor-derived malignancy was detected.

## 2. Case Report

A 62-year-old woman presented with a two-year history of progressive but asymptomatic thrombocytopenia and leukopenia in 2018. Bone marrow examination showed megakaryocyte dysplasia without increased blasts (myelodysplastic syndrome (MDS)-SLD) with a normal female karyotype. NGS testing revealed a biallelic SRSF2 mutation and a TET2 mutation.

She was managed with observation alone until 2021, when a repeat bone marrow examination was performed for progressive pancytopenia. The aspirate now showed trilineage dysplasia with 10%–15% blasts by morphology, and no ring sideroblasts (MDS-IB2). On karyotype, 16 of 20 metaphases showed isodicentric X chromosomes, and another 3 showed loss of X.

She was treated with 4 cycles of 5-azacitidine prior to transplantation. Interim bone marrow aspirate after 2 cycles showed reduction in blast count to less than 3%. In 2022, she received an allogeneic stem cell transplant from her sister, a 10/10 HLA-matched related donor. There was no family history of any hematologic disorder.

Reduced intensity conditioning consisted of fludarabine, busulfan, and total body irradiation. A total of 3 × 10^6^ CD34-positive stem cells were infused. Graft versus host disease prophylaxis consisted of anti-thymocyte globulin, methotrexate, and cyclosporine.

Posttransplant, our patient had ongoing transfusion-dependent pancytopenia. Day 98 bone marrow examination showed trilineage dysplasia, and chimerism showed 4% myeloid host cells concerning for an early disease relapse. She was treated with 5-azacitidine for 2 cycles and then underwent a donor lymphocyte infusion (DLI) of 1 × 10^7^ CD3+ cells/kg. She developed acute GVHD following this, thus limiting further DLI.

Another bone marrow examination was performed 26 months following the DLI (32 months posttransplant) for persistent bicytopenia. The aspirate showed a paucity of mature myeloid cells and megakaryocytes, with 10%–12% blasts (relapsed MDS-IB2 or early AML). FLT3 PCR testing was negative. Chimerism analysis continued to show > 98% donor cells in all leukocyte lineages (Table 1). Full karyotyping was performed, showing Monosomy 7 in 17 of 21 metaphases (45,XX,−7[17]/46,XX[4]), consistent with a donor-derived MDS.

**Figure 1:**
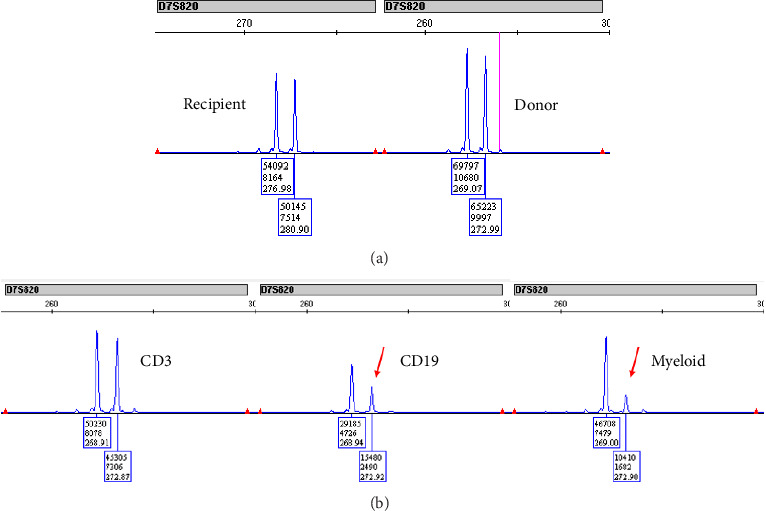
(a) Electropherogram from GeneMapper 6 showing the recipient (left) and donor (right) profiles for the chromosome 7 marker D7S820 prior to transplant. Note the difference in recipient fragment length (∼277 and 281 bp) versus donor fragment length (∼269 and ∼273 bp). (b) Electropherogram from GeneMapper 6 showing the apparent loss of chromosome 7 represented by the marker D7S820, primarily in the CD19 and myeloid cell lines. The CD3 cell line showed an apparently normal copy number of chromosome 7. Posttransplant fragment lengths match the donor profile in all cell lines.

Further inspection revealed a missing microsatellite site (D7S820) on the donor cells (Figure 1). Initially, this marker showed equal proportions of Sites 269 and 273 prior to DLI; on the current sample, there was a 4.4:1 ratio in the peaks at 269 to 273. This was consistent with the 17:4 ratio seen on conventional karyotyping. Re-examination of prior chimerism samples revealed a similar 3.6:1 ratio at 269:273 was at 20 months post-DLI (26 months posttransplant), 6 months prior to the diagnosis of donor-derived MDS. At that time, routine practice did not report changes in a single microsatellite marker, so the clinical team was not aware of this change. Her biopsy at that time was nondiagnostic for MDS both morphologically and immunophenotypically, so she continued with observation alone until the repeat biopsy 6 months later (32 months following allo-HSCT, 26 months following DLI) showed frank progression of her disease.

Our recipient has since started back on 5-azacitidine treatment. A search for an unrelated donor is underway. Her donor continues to have normal counts, but it was recommended that she undergo regular follow-up to monitor for the development of MDS.

## 3. Methods

Cell lines were magnetically isolated from the peripheral blood into CD3+ T cell, CD19+ B cell, and myeloid cell lines using a StemCell RoboSep-S and the EasySep HLA Chimerism Positive Selection kit. DNA was extracted from each cell line using the Qiagen QIAcube Connect extraction instrument, and the concentration was determined using a Qubit 3.0 fluorometer. PCR was completed using the AmpFLSTR Identifiler PCR Amplification kit following the manufacturer recommended protocol. The amplified samples were loaded onto the Applied Biosystems SeqStudio genetic analyzer to determine fragment lengths.

Data were imported into Thermo Fisher's GeneMapper 6 software for analysis. The sample was evaluated for the percentages of donor and recipient DNA using amelogenin locus and 15 DNA microsatellite markers (D2S1338, TPOX, D3S1358, FGA, D5S818, CSF1PO, D7S820, D8S1179, TH01, vWA, D13S317, D16S539, D18S51, D19S433, and D21S11).

## 4. Discussion

A 62-year-old woman with MDS-SLD, diagnosed in 2018, progressed to MDS-IB2 by 2021. Initial treatment with 5-azacitidine reduced her blast count, allowing her to undergo allo-HSCT using a graft from her HLA-matched sister. Despite achieving high donor chimerism, she experienced persistent pancytopenia and relapsed with donor-derived MDS characterized by monosomy 7. Interestingly, 20% of reported donor-derived leukemia patients have a chromosome 7 abnormality [4], which is also associated with therapy-related acute myeloid leukemia (AML) [9].

There are a variety of possible explanations for the rapid onset of donor-derived malignancy. The host microenvironment plays an active role in the growth of hematopoietic stem cell clones and, if affected by prior cytotoxic exposure or underlying acquired abnormalities, could allow for abnormal donor-derived clones to obtain a competitive advantage [10–12]. These clones may have already been present below the detection of routine screening. Preexisting clones may expand quickly when placed under the stress of engraftment as well as posttransplant immunosuppression [3, 12–14]. Alternatively, donor-derived malignant clones could develop de novo. The transplant process creates replicative stress with the need for rapid hematopoietic cell repopulation. This may exceed donor cell DNA repair capacity, increasing the risk of genomic instability. Resulting microsatellite or chromosomal alterations can result in rapid clonal selection and cytopenias [15]. Finally, recent studies indicate that subclonal expansions and “clonal sweeps” posttransplant may give the impression of accelerated disease progression in otherwise indolent disorders [16, 17].

This case underscores the clinical utility of lineage-specific chimerism analysis in the early detection of donor-derived MDS following allo-HSCT. Chimerism analysis is essential for identifying early genetic shifts indicative of disease recurrence or the development of donor-derived malignancies. Here, a patient with progressive MDS was treated with allo-HSCT from her sister. Despite achieving over 98% donor chimerism across all leukocyte lineages posttransplant, persistent pancytopenia and relapse consistent with MDS/AML were observed. Notably, a microsatellite marker imbalance at D7S820 on chromosome 7, correlating with monosomy 7 in donor-derived cells, was detected. This suggests that chimerism analysis can reveal early genetic anomalies, such as monosomy 7, which signal the onset of donor-derived MDS, even when global donor chimerism remains high.

Retrospective analysis revealed the imbalance in the D7S820 microsatellite marker ratio at least six months before the clinical diagnosis of donor-derived MDS. However, because single-marker changes are not routinely flagged in standard chimerism reports, this early signal went unrecognized, representing a missed opportunity for earlier clinical intervention. This case highlights the critical need to incorporate a detailed chimerism marker analysis into posttransplant monitoring to uncover subtle but meaningful genetic changes, such as marker-specific imbalances, that may indicate clonal evolution within donor cells.

This case underscores the need for detailed chimerism marker analysis in posttransplant follow-up to enable the timely identification of donor-derived malignancies and improve patient prognosis.

## Figures and Tables

**Table 1:** Recipient chimerism analysis over time.

Time post-allo-HSCT	CD3+ cells	CD19+ cells	Myeloid cells
Day 62	75% donor	95% donor	> 98% donor
Day 98	80% donor	96% donor	**96%** donor
Day 171 (pre-DLI)	59% donor	> 98% donor	> 98% donor
Month 26	> 98% donor	> 98% donor	> 98% donor
Month 32	> 98% donor	> 98% donor	> 98% donor

*Note:* The patient received 2 cycles of 5-azacitidine between Day 98 and Day 171 chimerism analysis due to relapsing disease. Following DLI at 6 months, donor-derived cells predominated, confirming second MDS diagnosis was donor-derived. The bold values highlight abnormal results.

## Data Availability

The data supporting the findings of this case report are available within the article.
